# EPDR1 Links Fibroblast Dysfunction to Disease Severity in Idiopathic Pulmonary Fibrosis

**DOI:** 10.3390/cells14191515

**Published:** 2025-09-28

**Authors:** Jong-Uk Lee, Seung-Lee Park, Min Kyung Kim, Eunjeong Seo, Hun-Gyu Hwang, Jung Hyun Kim, Hun Soo Chang, Choon-Sik Park

**Affiliations:** 1 Department of Interdisciplinary Program in Biomedical Science Major, Soonchunhyang Graduate School, Bucheon 14584, Republic of Korea; lajoal@nate.com (J.-U.L.);; 2 Department of Internal Medicine, Soonchunhyang University Bucheon Hospital, Bucheon 14584, Republic of Korea; 3 Department of Internal Medicine, Soonchunhyang University Seoul Hospital, Seoul 04401, Republic of Korea; 4 Department of Microbiology, College of Medicine, Soonchunhyang University, Cheonan 33151, Republic of Korea

**Keywords:** EPDR1, idiopathic pulmonary fibrosis, lysosome, fibroblasts, autophagy, cellular senescence

## Abstract

**Highlights:**

**What are the main findings?**

- EPDR1 is significantly upregulated in IPF lung fibroblasts, tissues, BALF, and serum, and it is associated with poor prognosis.
- EPDR1 knockdown restores lysosomal acidification, improves autophagic flux, and reduces cellular senescence in IPF fibroblasts.

**What is the implication of the main finding?**

- EPDR1 contributes to lysosomal dysfunction and fibroblast senescence, which are key pathogenic features of IPF.
- EPDR1 may serve as a novel biomarker and potential therapeutic target in idiopathic pulmonary fibrosis.

**Abstract:**

Idiopathic pulmonary fibrosis (IPF) is a progressive lung disease characterized by aberrant fibroblast activation, lysosomal dysfunction, and cellular senescence. Transcriptomic analyses have identified ependymin-related 1 (EPDR1) as a fibroblast-enriched gene in IPF, but its biological function remains unclear. EPDR1 expression was assessed in lung fibroblasts, lung tissues, bronchoalveolar lavage fluid (BALF), and serum from IPF patients and controls using qPCR, Western blotting, ELISA, and immunohistochemistry. Lysosomal function, autophagic flux, and senescence markers were analyzed in primary fibroblasts following siRNA-mediated EPDR1 knockdown. EPDR1 was significantly upregulated in IPF-derived fibroblasts and localized to fibrotic regions enriched with α-SMA^+^, COL1A1^+^, and FN1^+^ myofibroblasts of IPF-derived lung tissues. EPDR1 levels were markedly elevated in the BALF and serum of IPF patients and correlated with increased mortality. IPF fibroblasts exhibited reduced lysosomal acidification and impaired autophagic flux, indicated by p62 and LC3B accumulation. EPDR1 knockdown restored lysosomal function; enhanced autophagic degradation; and reduced senescence markers, including p21, p16, and SA-β-gal activity. EPDR1 drives lysosomal dysfunction and fibroblast senescence in IPF. Its elevated expression in lung tissue and biological fluids, together with its association with prognosis, highlights EPDR1 as a potential biomarker and therapeutic target in IPF.

## 1. Introduction

Idiopathic pulmonary fibrosis (IPF) is initiated by alveolar epithelial cell damage and hyperplasia followed by the development of a profibrotic environment in which collagen-producing myofibroblasts accumulate through epithelial-to-mesenchymal transition (EMT) and fibroblast-to-myofibroblast transition (FMT) [1]. The typical histological finding is the presence of fibroblast foci: collections of fibroblasts and myofibroblasts actively producing the extracellular matrix (ECM). Thus, the dysregulation of fibroblasts is one of the main mechanisms behind the rapid progression of fibrosis with intractable course [2]. Fibroblasts from IPF lungs exhibit several differences in phenotypes compared with those isolated from non-fibrotic lungs: resistance to apoptosis, enhanced myofibroblastic differentiation, and accelerated cellular senescence [1]. Thus, mesenchymal cells in IPF contribute to disease pathogenesis through early acquisition of the fibrogenic phenotype during the differentiation process.

To define genes responsible for these proporties, several transcriptome studies over the whole genome have been conducted, revealing additional candidate genes related with the dysregulated fibroblasts [3,4,5,6]. Recently, we identified 15 genes differentially expressed more than 10 times by IPF lung tissue—derived fibroblasts compared with control fibroblasts [7]. Among them, mRNA expression of the ependymin-related protein 1 (*EPDR1*) gene was more than twofold higher in those of IPF than the control lungs. At the single-cell level using single-cell RNA-sequencing (scRNA-seq), mesenchymal cells obtained from IPF expressed high levels of EPDR1 when comparing those from control lungs [8]. Despite the ubiquitous presence of EPDR1 in multiple organs, the molecular functions have remained unknown, except the pathophysiological functions of EPDR1 in myofibroblast contractility of Dupuytren’s disease [9].

EPDR1 (ependymin-related protein 1) is a lysosomal/endosomal protein that plays a role in intracellular trafficking and ECM regulation. Subcellular fractionation studies in mammalian brain tissue have demonstrated that EPDR1 co-fractionates with classical lysosomal markers, supporting its localization within lysosomal compartments [10]. In vitro imaging further confirms that EPDR1 tagged with mCherry co-localizes with GFP-tagged lysosomal markers, indicating robust lysosomal enrichment [11]. In addition to its intracellular localization, EPDR1 is secreted into the extracellular milieu and can be re-internalized via endocytic pathways, subsequently trafficking back to lysosomes [11]. Although fibroblasts are known to form phagolysosomes by endocytosing extracellular debris, EPDR1 localization within lung fibroblast-derived phagolysosomes has not yet been directly visualized.

EPDR1 is secreted by senescent fibroblasts and has been identified as a senescence-associated secretory factor. In skin fibroblasts, age-related upregulation of EPDR1 promotes paracrine suppression of collagen type I (COL1A1) synthesis and upregulation of matrix-degrading enzymes such as MMP1 and MMP3 in neighboring cells, indicating a potential role in negatively regulating ECM deposition [12]. Moreover, EPDR1 has been associated with fibroblast contractility, such as in a genetic study in Dupuytren’s disease (a localized fibrotic disorder) that identified EPDR1 as a susceptibility gene modulating myofibroblast contractile activity [9]. At the molecular level, EPDR1 binds anionic lipids such as bis-monoacylglycerol-phosphate (BMP) and ganglioside GM1 in an acidic environment, suggesting a possible function as a lysosomal lipid transporter or activator, similar to saposin-like proteins [13,14]. These findings collectively suggest that EPDR1 may regulate fibrotic remodeling by modulating fibroblast matrix secretion and degradation, as well as lysosomal lipid homeostasis. However, its functional role in lung fibroblasts and IPF pathogenesis remains to be elucidated.

This prompted us to reveal the relation of EPDR1 with fibrogenesis of IPF. In the present study, we compared the expression of the EPDR1 protein and mRNA levels by lung-derived fibroblasts between IPF and controls, as well as its localization in lung tissues obtained from IPF patients and controls. In addition, we measured EPDR1 protein levels in bronchoalveolar lavage fluid (BALF) and serum between IPF and controls to relate with clinical impacts. Furthermore, we compared intracellular lysosomal acidification in lung-derived fibroblasts between IPF patients and controls and examined the effect of EPDR1 knockdown on phagolysosomal function.

## 2. Materials and Methods

### 2.1. Study Subjects

Chest X-rays and high-resolution chest computed tomography (HRCT) were performed for all subjects, along with pulmonary function testing focused on forced vital capacity (FVC) and diffusing capacity of the lung for carbon monoxide (DLco) values. BALF, serum, and lung tissue samples were obtained from the biobank of Soonchunhyang University Hospital in Bucheon, Republic of Korea, with approval from the Institutional Review Board (IRB) of Soonchunhyang University (SCH 2025-02-006). Control BALF samples were obtained from medical students and hospital staff with the approval of the hospital Ethics Committee (IRB No: SCHBC 2015–08–025–005). Informed written consent to participate was obtained from each subject. IPF was diagnosed by identifying usual interstitial pneumonia patterns in histopathological samples or through multidisciplinary discussion without the need for a lung biopsy, as outlined in the 2011 and 2018 guidelines [15,16]. None of the patients diagnosed with IPF exhibited any signs of underlying collagen vascular diseases, as confirmed by laboratory tests and clinical symptoms. Controls did not show any respiratory symptoms, as confirmed by a screening questionnaire, and had forced expiratory volume in 1 s (FEV1) and FVC levels above 80%, along with normal chest radiographs.

### 2.2. Culture of Primary Human Fibroblasts Obtained from Biopsy Specimens

Primary lung fibroblasts were isolated from surgical lung tissues obtained from 10 patients diagnosed with IPF and 10 control individuals who had undergone resection for stage I or II lung cancer, as performed previously [7]. Detailed clinical data are available in Table S1. In brief, lung tissue samples were mechanically minced and cultured in 150 cm^2^ flasks using tissue culture medium (TCM) composed of Dulbecco’s modified Eagle’s medium (DMEM; Lonza, Walkersville, MD, USA), supplemented with 10% fetal bovine serum (FBS; Thermo Fisher Scientific, Rockford, IL, USA), 2 mM L-glutamine, and 1% penicillin–streptomycin–amphotericin solution (Lonza). Cultures were maintained at 37 °C with 5% CO_2_ until adherent fibroblast populations were established. Subculturing continued until passage 4 to ensure fibroblast purity, after which cells were cryopreserved at −170 °C. For experimental use, fibroblasts at passage 5 (4 × 10^5^ cells per 10 mL TCM) were seeded into 100 cm^2^ culture dishes. Once cultures reached approximately 90% confluency, cells were rinsed twice with phosphate-buffered saline (PBS; Thermo Fisher Scientific) and harvested for RNA and protein extraction. Total RNA was isolated using TRI Reagent (Ambion, Carlsbad, CA, USA), while protein lysates were prepared in RIPA buffer. Protein concentrations were determined via BCA assay (Thermo Fisher Scientific). To verify the identity and purity of the cultured fibroblasts, immunoblotting was performed using cell-type-specific markers (Figure S1).

### 2.3. Silencing of EPDR1 via Electroporation of siRNA

In order to knock down EPDR1, 100 nM negative control (SCR) (SN-1003, Bioneer, Daejeon, Republic of Korea) and siRNA (sense strand sequence: 5′-GGAUCUUCCUCUCAACAUU-3′; 54749-3, Bioneer, Republic of Korea) were transfected into human primary lung fibroblasts via electroporation using the Lonza 4D-Nucleofector with the SE Cell Line 4D-Nucleofector™ X Kit (V4XC-1024, Lonza, Cologne, Germany), following the manufacturer’s protocol. The cells were incubated for 24 h prior to each experiment.

### 2.4. Quantitative Polymerase Chain Reaction (qPCR)

Total RNA was extracted using TRIzol reagent following the manufacturer’s protocol (Invitrogen, Carlsbad, CA, USA). For cDNA synthesis, 3 μg of RNA was suspended in DEPC-treated water and incubated at 65 °C for 5 min with 0.5 μg of oligo (dT) primers and 10 mM dNTPs. The mixture was then rapidly cooled on ice. PCR amplification was conducted for 30 cycles, comprising an initial denaturation at 94 °C for 5 min, followed by repeated steps of 94 °C for 30 s, 60 °C for 30 s, and 72 °C for 30 s. A final extension step was carried out at 72 °C for 7 min. The primer sequences used in this study are listed in Table S2. qPCR was conducted on the StepOne™ Real-Time PCR platform (Applied Biosystems, Foster City, CA, USA). Each 20 μL reaction included 1 μg of cDNA, 1 μL of 10 pmol forward and reverse primers, and 10 μL of 2× SYBR Green PCR Master Mix (Applied Biosystems, Foster city, CA, USA). The reaction was carried out in a two-step procedure: denaturation at 95 °C for 15 s and 60 °C for 1 min, and melting at 95 °C for 15 s, 60 °C for 1 min, and 95 °C for 15 s. Data were analyzed using the 2^−ΔΔCT^ method [17] and presented as the relative fold change with normalization to β-actin.

### 2.5. Western Blot Analyses

Protein extracts (30 μg per sample) prepared using RIPA buffer were resolved on a 12.5% SDS-PAGE gel and subsequently transferred onto polyvinylidene difluoride (PVDF) membranes (Millipore, Billerica, MA, USA). Membranes were then blocked for 1 h at room temperature in Tris-buffered saline with 0.1% Tween 20 (TBST) containing 5% skim milk (BD Biosciences, Sparks, MD, USA). Following blocking, the membranes were incubated with primary antibodies: rabbit polyclonal anti-EPDR1 (1:1000; Abcam, Cambridge, UK), mouse monoclonal anti-LC3B (1:1000; Santa Cruz Biotechnology, Dallas, TX, USA), mouse monoclonal anti-human p62 (1:1000; Santa Cruz Biotechnology, Santa Cruz, CA, USA), rabbit polyclonal anti-human p21 (1:1000; Abcam, Cambridge, UK), rabbit monoclonal anti-human p16 (1:1000; Cell Signaling Technology, Danvers, MA, USA), rabbit polyclonal anti-human α-smooth muscle actin (α-SMA) (1:2000; Abcam, Cambridge, UK), rabbit polyclonal anti-human COL1A1 (1:1000; Abcam, Cambridge, UK), rabbit polyclonal anti-human fibronectin (FN1) (1:2000; Abcam, Cambridge, UK), and mouse monoclonal anti-human β-actin (1:50,000; Sigma-Aldrich). Following multiple washes with TBST, membranes were treated with horseradish peroxidase (HRP)-conjugated goat anti-mouse or anti-rabbit IgG secondary antibodies (GenDEPOT, Baker, TX, USA). Signal detection was performed using chemiluminescent substrates (Thermo Fisher Scientific and Bio-Rad, Berkeley, CA, USA) and visualized on the ChemiDoc™ Touch Imaging System (Bio-Rad). Band intensities were quantified, normalized against β-actin, and reported as arbitrary units (AU).

### 2.6. Measurement of EPDR1 Protein in BALF

BAL was carried out at the time of enrollment, targeting the most affected lung segments identified on HRCT in patients, or the right middle lobe in healthy controls, following established protocols [18]. Total cell numbers were determined using a hemocytometer. Supernatants were separated using centrifugation and were stored at −80 °C. Differential count of 500 cells was performed on slides of BALF cells prepared by cytocentrifugation (500 G, 5 min) and stained with Diff-Quik solution. Levels of EPDR1 protein in BALF and serum were quantified using a commercially available ELISA kit (MyBioSource, San Diego, CA, USA), following the supplier’s instructions. The assay’s detection threshold was 1 ng/mL, and any measurements below this limit were recorded as 0 ng/mL.

### 2.7. Immunofluorescence (IF) Double Stain of EPDR1 with α-SMA, COL1A1, and FN1

Formalin-fixed, paraffin-embedded lung tissues from IPF patients and controls were sectioned at a thickness of 4 μm, followed by deparaffinization, rehydration, and staining with hematoxylin and eosin (H&E). The sections were incubated in a Fc receptor blocking agent (FC blocker, InnovexBiosciences, Richmond, CA, USA) for 30 min, treated with 5% BSA in TBS for 1 h to block non-specific binding, and then incubated with polyclonal rabbit anti-human EPDR1 antibody (1:100, Abcam, Cambridge, MA, USA), monoclonal mouse anti-human α-SMA antibody (1:100, Santa Cruz Biotechnology), monoclonal mouse anti-human COL1A1 (1:100, BIOSS, Boston, MA, USA), and monoclonal mouse anti-human FN1 (1:100, Abcam) in 5% BSA overnight at 4 °C. Following washes with 1× TBS, tissue sections were treated with fluorescently labeled secondary antibodies—FITC-conjugated goat anti-rabbit (1:1000, Abcam) or PE-conjugated goat anti-mouse (1:1000, Abcam). Nuclear counterstaining was performed using DAPI (Invitrogen, Carlsbad, CA, USA). Fluorescent images were acquired with a confocal laser scanning microscope (Leica, Wetzlar, Germany), and images were acquired using a Leica system. Image processing and editing were performed using ZEN 2009 (Light Edition, Zeiss, Oberkochen, Germany).

### 2.8. Analysis of Lysosomal Acidity and EPDR1 Colocalization Using LysoTracker

Primary lung fibroblasts (8 × 10^4^ cells) were seeded onto 18 × 18 mm glass coverslips and cultured for 24 h. Rapamycin (S553210; Sigma-Aldrich, St. Louis, MO, USA) was applied to the cells, followed by staining with LysoTracker Red DND-99 (L7528; Thermo Fisher Scientific, Waltham, MA, USA), as previously described [19,20]. Specifically, cells were stained with 1 µg/mL Hoechst 33342 (H3570; Thermo Fisher Scientific, Waltham, MA, USA) for 1 h at 37 °C, followed by incubation with 100 nM LysoTracker Red DND-99 in DMEM for 1 h at 37 °C. After washing, confocal imaging was performed using a Leica Microsystems microscope (Wetzlar, Germany) at 100× magnification. All images were acquired under identical settings: pinhole set to 3, gain adjusted to 800, and fluorescence signals from LysoTracker Red DND-99 (excitation 577 nm, emission 590 nm) and Hoechst 33342 (excitation 350 nm, emission 461 nm) were detected. Twenty fields were captured per sample to ensure representative sampling. LysoTracker fluorescence intensity was measured using ImageJ software (NIH, Bethesda, MD, USA; version 1.54p). To correct for inter-experimental variability, a rapamycin dose–response curve (0–10 μM) was generated in MRC5 cells and used as a normalization reference, such that fluorescence intensities from experimental samples were expressed as rapamycin-equivalent doses.

To assess the subcellular localization of EPDR1, immunofluorescence staining was additionally performed in LysoTracker-labeled fibroblasts using a FITC-conjugated anti-EPDR1 antibody. Colocalization between EPDR1 and lysosomes was analyzed using the Coloc2 plugin in Fiji (https://fiji.sc/, accessed on 21 September 2025), following the methodology described by Shakhov et al. (2022) [21]. Confocal images were processed using ZEN 2009 (Light Edition, Zeiss, Oberkochen, Germany). After uniform background subtraction, cytoplasmic regions of interest (ROIs) were manually selected to minimize background interference. Manders’ coefficients M1 and M2 were calculated to quantify fluorescence overlap, ranging from 0 (no colocalization) to 1 (complete colocalization). Six images were analyzed per group.

### 2.9. Senescence-Associated β-Galactosidase (SA-β-Gal) Activity

Primary lung fibroblasts (8 × 10^4^ cells) were seeded in 12-well plates and cultured for 24 h prior to staining. SA-β-gal staining was conducted using the Cellular Senescence Assay Kit (CBA-230; Cell Biolabs Inc., San Diego, CA, USA) following the manufacturer’s instructions. For each sample, six random fields were imaged under a light microscope at 100× magnification, as previously described [22].

### 2.10. Statistical Analysis

All statistical analyses were conducted utilizing SPSS software (version 22; SPSS Inc., Chicago, IL, USA). The normality of the data distribution was assessed via the Shapiro–Wilk test. Based on the distribution characteristics, continuous variables were analyzed using either Student’s t-test or the Mann–Whitney U test. Categorical variables were examined employing the chi-square test or Fisher’s exact test. Associations between EPDR1 levels and clinical parameters were evaluated by Spearman’s correlation analysis. Receiver operating characteristic (ROC) curve analysis was performed to determine cut-off values of EPDR1 concentrations. One cut-off was derived to distinguish IPF patients from controls, based on the point with the highest sensitivity and specificity. In addition, the optimal cut-off value for survival analysis was determined using Youden’s index. The survival cut-off was subsequently applied for Kaplan–Meier analysis and Cox proportional hazards regression. Comparisons of the area under the curve values were conducted using Z-tests in MedCalc software (version 12.2.1.0; MedCalc Software, Ostend, Belgium) [23]. Kaplan–Meier analysis with the log-rank test was used to evaluate differences in survival between groups. To identify independent prognostic factors, Cox proportional hazards regression with backward stepwise selection was applied. To adjust for age differences between groups when comparing senescence markers, a nonparametric analysis of covariance (Quade’s ANCOVA) was applied using age as a covariate. Skewed data are presented as median value with the 25th and 75th percentile, and normally distributed data are expressed as mean ± standard error of the mean. *p*-values < 0.05 were considered significant.

## 3. Results

### 3.1. Clinical Characteristics of the Study Groups

Since elevated EPDR1 mRNA levels were identified in a prior transcriptomic study of IPF fibroblasts (*n* = 8) compared with controls (*n* = 4) [9], we validated this finding by measuring EPDR1 mRNA (qPCR) and protein (Western blot) levels in an expanded cohort of fibroblasts (10 IPF and 10 controls). The clinical characteristics of the 20 subjects are presented in Table S1. There was no difference of age, sex, and smoking status between the two groups except lower FVC and DL_CO_ in IPF. BALF and serum samples were obtained from patients with IPF and controls, and their clinical characteristics are summarized in Table 1. In the BALF, IPF patients (*n* = 131) showed significantly higher total cell counts and elevated proportions of macrophages, neutrophils, eosinophils, and lymphocytes compared with controls (*n* = 52) (*p* < 0.05). Pulmonary function, including FVC and DL_CO_ (% predicted), was also significantly reduced in the IPF group (*p* < 0.05). Among the study subjects, serum samples were obtained from 114 IPF patients and 45 controls.

### 3.2. Comparison of EPDR1 Expression in Lung-Tissue-Derived Fibroblasts from IPF Patients and Controls

**Figure 1:**
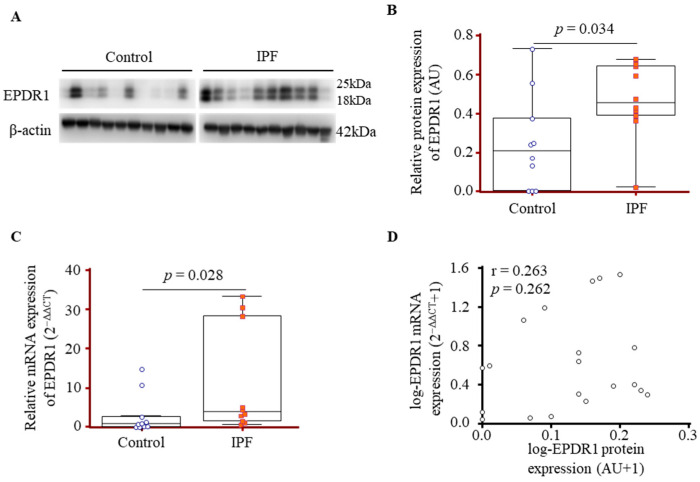
EPDR1 gene and protein expression in lung fibroblasts from IPF patients and controls. (**A**) Western blot analysis was performed on fibroblasts derived from the lung tissue of 10 IPF patients and 10 controls. (**B**) Densitometric quantification of EPDR1 band intensity normalized to β-actin. (**C**) EPDR1 mRNA expression in the same samples was assessed by qPCR, normalized to β-actin, and analyzed using the 2^−ΔΔCT^ method. (**D**) Correlation between EPDR1 expression measured by Western blot (AU) and qPCR (2^−ΔΔCT^). Data were log_10_(x + 1)-transformed prior to Spearman analysis to minimize skew and avoid negative values. Data are presented as median values with interquartile ranges (25th–75th percentile).

EPDR1 expression was examined in primary lung fibroblasts isolated from control and IPF patients using both protein and mRNA analyses. Western blot analysis revealed that EPDR1 protein levels were markedly higher in IPF lung fibroblasts compared to controls [0.45 (0.38–0.64) vs. 0.20 (0.01–0.42) AU, *p* = 0.034; Figure 1A,B]. In line with this finding, qPCR analysis showed significantly elevated EPDR1 mRNA expression in IPF fibroblasts relative to controls [3.95 (1.47–28.93) 2^−(ΔΔCT)^ vs. 1.01 (0.19–4.80) 2^−(ΔΔCT)^, *p* = 0.028; Figure 1C]. A positive trend was observed between EPDR1 mRNA and protein levels, but the correlation was not statistically significant (r = 0.263, *p* = 0.262; Figure 1D). 

### 3.3. IF Localization of EPDR1 Protein in Lung Tissues

**Figure 2:**
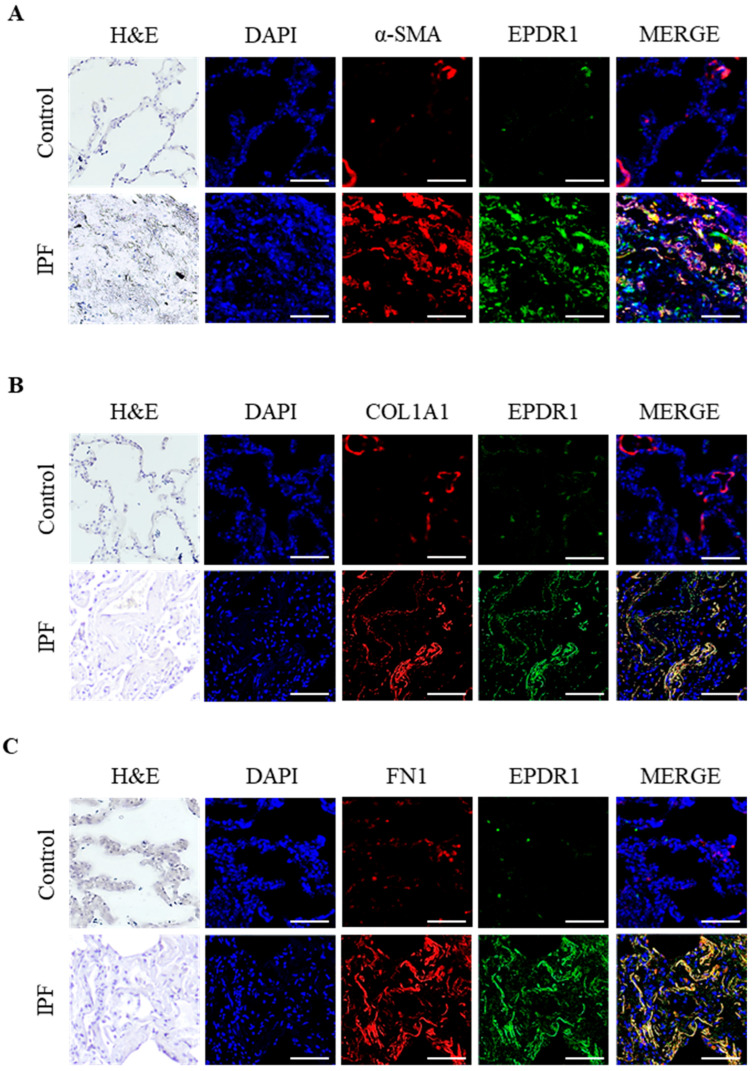
Representative IF images of lung tissues from IPF patients and controls. Sections were stained for EPDR1 with (**A**) α-smooth muscle actin (α-SMA), (**B**) type I collagen (COL1A1), or (**C**) fibronectin (FN1); nuclei were counterstained with DAPI. Images were acquired at ×400 magnification. Scale bar = 200 μm.

To localize EPDR1 expression in lung tissues and compare its distribution between IPF and control lungs, IF double staining of EPDR1 with α-SMA, COL1A1, and FN1 proteins was performed. In control lung tissues, α-SMA and EPDR1 staining were barely detectable on the alveolar walls and interstitial areas, indicating low basal expression (Figure 2A). In contrast, in IPF lung tissues, EPDR1 was strongly expressed and co-localized with α-SMA–positive cells in the fibrotic interstitial regions. Similarly, EPDR1 protein co-localized with COL1A1 and FN1 proteins in areas with fibroblast accumulation and collagen deposition, as confirmed by H&E staining (Figure 2B,C).

### 3.4. Comparison of EPDR1 Protein Levels in BALF and Serum Between IPF Patients and Controls, and Their Association with Survival in IPF

**Figure 3:**
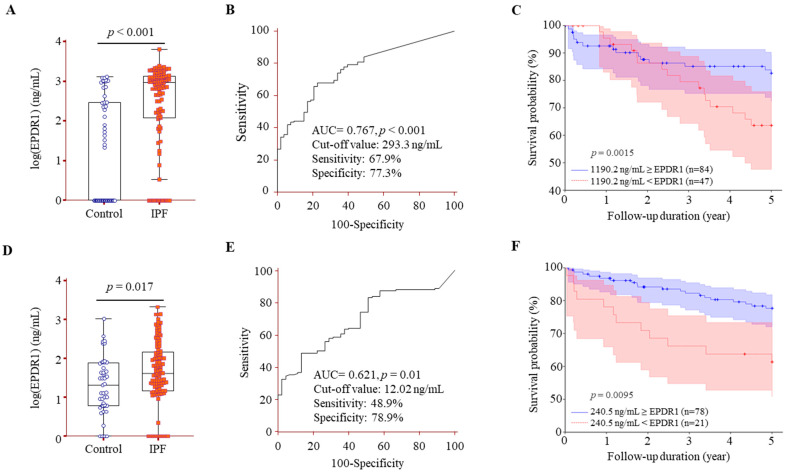
EPDR1 protein concentrations in BALF and serum: comparisons, diagnostic performance, and survival analysis. (**A**) EPDR1 levels in BALF from controls (*n* = 52) and IPF patients (*n* = 131). (**B**) ROC curve analysis of BALF EPDR1 concentrations for discriminating IPF from controls. (**C**) Kaplan–Meier survival analysis stratified by BALF EPDR1 levels (dotted line: >1190.2 ng/mL, solid line: ≤1190.2 ng/mL). (**D**) EPDR1 levels in serum from controls *(n* = 45) and IPF patients (*n* = 114). (**E**) ROC curve analysis of serum EPDR1 concentrations for discriminating IPF from controls. (**F**) Kaplan–Meier survival analysis stratified by serum EPDR1 levels (dotted line: ≥240.5 ng/mL, solid line: <240.5 ng/mL). Data are shown as medians with interquartile ranges (25th–75th percentiles).

EPDR1 protein was detected in BALF from 24 of 52 controls and from 108 of 131 IPF. The levels were significantly higher in the IPF [938.6 (113.4–1302.2) ng/mL] compared with those in the control [0 (0–288.5) ng/mL, *p* < 0.001] (Figure 3A). An ROC curve of the EPDR1 protein concentrations demonstrated a clear distinction between the IPF and control with an area under the curve (AUC) of 0.767 (*p* < 0.001), and the cut-off value of 293.3 ng/mL showed 77.3% specificity and 67.9% sensitivity (Figure 3B). Using Youden’s index, the optimal cut-off value of EPDR1 in BALF was determined to be 1190.2 ng/mL, which yielded the highest discriminatory performance (C-index = 0.653, *p* = 0.0015). The univariate analysis revealed that patients with EPDR1 levels ≥ 1190.2 ng/mL had a significantly increased risk of mortality compared to those with lower levels [hazard ratio (HR) = 2.19; 95% confidence interval (CI): 1.08–4.43; *p* = 0.029] (Table 2). In the multivariable analysis, which included FVC and DL_CO_ as covariates, high levels of EPDR1 remained independently associated with a higher risk of mortality (HR = 2.21; 95% CI: 1.04–4.67; *p* = 0.038). Kaplan–Meier analysis of 120 subjects followed for up to 5 years revealed significantly lower survival in those with EPDR1 levels > 1190.2 ng/mL compared to those with lower levels via the log-rank test (*p* = 0.0015, Figure 3C).

EPDR1 was detected in serum from 40/45 controls and 97/114 IPF patients and was significantly elevated in IPF compared with controls ([40.2 (13.6–144.1) ng/mL] vs. [20.2 (5.0–59.7) ng/mL], *p* = 0.017) (Figure 3D). Serum EPDR1 modestly distinguished IPF from controls (AUC = 0.621, *p* = 0.01), with an optimal cut-off of 12.02 ng/mL yielding 78.9% specificity and 48.9% sensitivity (Figure 3E). Serum EPDR1 was not a statistically significant predictor of mortality (C-Index = 0.588, *p* = 0.199); however, a provisional threshold of 240.5 ng/mL was identified on the basis of data-driven stratification: patients with serum EPDR1 ≥ 240.5 ng/mL had significantly worse survival in univariate analysis (HR = 2.65; 95% CI: 1.29–5.45; *p* = 0.008), and the association remained significant after adjusting for FVC and DL_CO_ in multivariable analysis (HR = 2.61; 95% CI: 1.13–5.95; *p* = 0.034) (Table 2). Kaplan–Meier survival analysis further confirmed that patients above this threshold had significantly poorer outcomes compared to those below it (log-rank *p* = 0.0095, Figure 3F).

### 3.5. Lysosomal Localization and Activity Changes Associated with EPDR1 in IPF Fibroblasts

**Figure 4:**
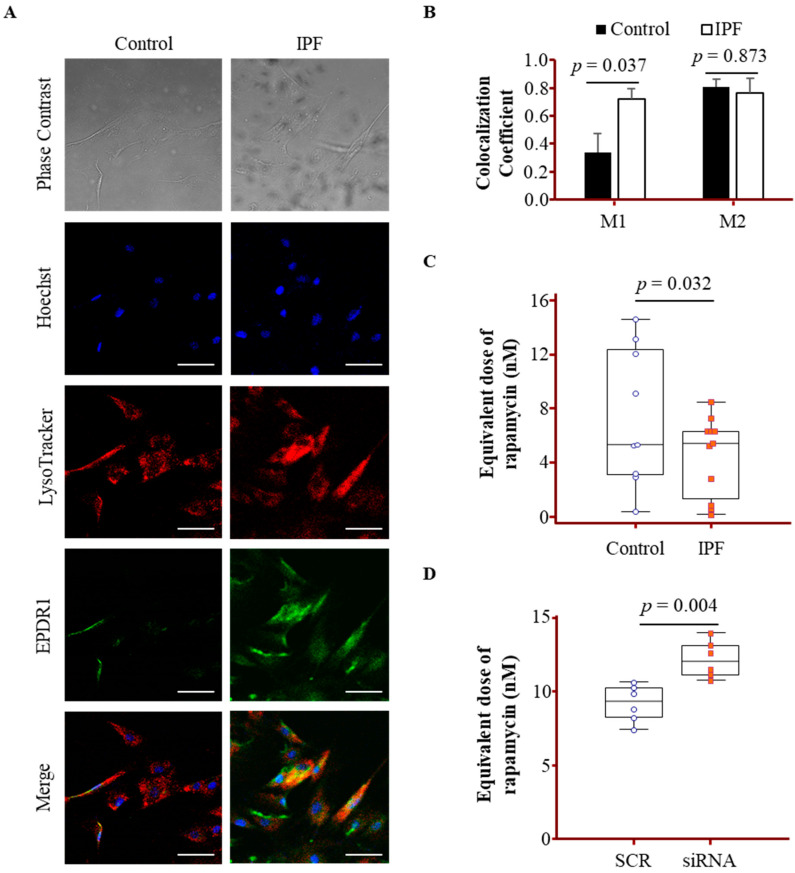
IF staining of EPDR1 and lysosomes, as well as the assessment of lysosomal activity in fibroblasts derived from IPF and control lungs. Primary lung fibroblasts (8 × 10^4^ cells) were seeded on glass coverslips, stained with Hoechst 33342 (nuclei, blue), LysoTracker Red DND-99 (lysosomes, red), and anti-EPDR1 antibody (green), and imaged by confocal microscopy at ×400 magnification, Scale bar = 50 μm. (**A**) Representative IF images of control and IPF fibroblasts. (**B**) Quantification of EPDR1 colocalization with lysosomes. Fluorescence images were analyzed using the Coloc2 plugin in Fiji. Manders’ coefficient M1 represents the fraction of lysosomal signal overlapping with EPDR1, and M2 represents the fraction of EPDR1 signal overlapping with lysosomes. Six images were analyzed per group. Data are presented as mean ± SEM. (**C**) Lysosomal acidity quantified as rapamycin-equivalent dose (nM) based on LysoTracker fluorescence in fibroblasts from controls (*n* = 10) and IPF patients (*n* = 10). (**D**) Lysosomal activity in six fibroblasts transfected with negative control siRNA (SCR, *n* = 6) or EPDR1-targeting siRNA (*n* = 6). Data are shown as medians with interquartile ranges.

To assess whether lysosomal function is altered in IPF fibroblasts, we first conducted LysoTracker staining. Lysosomal involvement was further examined through IF analysis, which revealed partial co-localization of EPDR1 with LysoTracker-positive compartments in both control and IPF fibroblasts (Figure 4A). Quantitative colocalization analysis using Manders’ coefficients (Figure 4B) showed that the fraction of lysosomal signal overlapping with EPDR1 (M1) was significantly higher in IPF fibroblasts (0.72 ± 0.07) compared to controls (0.34 ± 0.13), indicating enhanced EPDR1 association with lysosomes in IPF. M2 values, representing the fraction of EPDR1 signal overlapping with lysosomes, were 0.81 ± 0.06 in controls and 0.76 ± 0.10 in IPF fibroblasts. EPDR1 signal intensity was markedly higher in IPF fibroblasts, supporting its lysosomal localization. Lysosomal function was subsequently inferred by quantifying LysoTracker fluorescence intensity and applying a calibration curve generated using MRC-5 treated with defined concentrations of rapamycin. This analysis revealed a significantly higher rapamycin-equivalent dose in control fibroblasts [*n* = 10, 6.31 (3.13–12.32) nM] than in IPF fibroblasts [*n* = 10, 5.35 (0.77–6.29) nM, *p* = 0.032], indicating reduced lysosomal activity in the IPF group (Figure 4C). To evaluate the role of EPDR1 in lysosomal function, siRNA-mediated knockdown of EPDR1 was performed in fibroblasts. Lysosomal activity was significantly increased in EPDR1-silenced cells [*n* = 6, 12.04 (11.02–13.31) nM] compared to SCR-treated controls [*n* = 6, 9.31 (8.03–10.32), *p* = 0.004], suggesting a regulatory role for EPDR1 (Figure 4D).

### 3.6. Increased Expression of Autophagy and Senescence Markers in IPF Lung Fibroblasts

**Figure 5:**
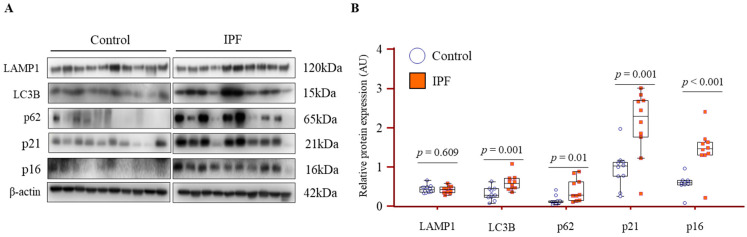
Expression of autophagy and senescence markers in fibroblasts derived from IPF and control lungs. (**A**) Western blot analysis of EPDR1, lysosomal marker (LAMP1), autophagy markers (LC3B, p62), and senescence markers (p21, p16) in primary lung fibroblasts from controls (*n* = 10) and IPF patients (*n* = 10). (**B**) Quantification of protein levels normalized to β-actin. Group comparisons were analyzed using Quade’s ANCOVA with age as a covariate. Data are presented as mean ± SEM.

Primary lung fibroblasts derived from IPF patients exhibited significantly higher levels of LC3B, p62, p21, and p16 compared to controls, as confirmed by Western blot analysis (Figure 5A). Quantification of the normalized protein expression levels further demonstrated these significant differences (Figure 5B), with *p*-values adjusted for age using a nonparametric ANCOVA, indicating that the observed changes are independent of age. In contrast, the expression of LAMP1, a marker of lysosomal membranes, showed no significant difference between the groups, suggesting that lysosomal abundance remains largely unchanged in IPF fibroblasts. To evaluate whether lysosome-related gene expression was altered at the mRNA level, qPCR analysis was performed in fibroblasts from controls (*n* = 10) and IPF patients (*n* = 10). The analyzed gene panel included lysosomal acidification machinery (ATP6V1A, ATP6V1B2, ATP6V0C, ATP6V0D1), lysosomal proteases (CTSB, CTSD), and the transcriptional regulator TFEB. No significant differences in transcript levels were detected between IPF and control fibroblasts (Figure S2).

### 3.7. EPDR1 Knockdown Attenuated Autophagy and Senescence Marker Expression in IPF Fibroblasts

**Figure 6:**
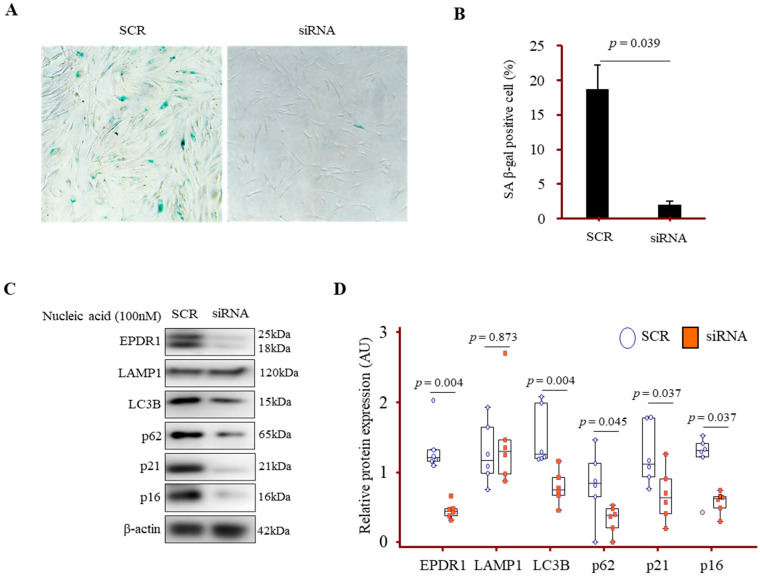
Effect of EPDR1 knockdown on autophagy and senescence marker expression in fibroblasts derived from IPF lungs. Primary lung fibroblasts were transfected with scramble (SCR) or EPDR1 siRNA. (**A**) Representative images of SA-β-gal staining in fibroblasts transfected with SCR or EPDR1 siRNA. (**B**) Quantification of SA-β-gal staining in fibroblasts transfected with SCR or EPDR1 siRNA. Quantification was performed using primary fibroblasts from three different patients. For each sample, six randomly selected fields were imaged under a light microscope at 100× magnification. (**C**) Western blot analysis of EPDR1, lysosomal marker (LAMP1), autophagy markers (LC3B, p62), and senescence markers (p21, p16) in six fibroblasts transfected with SCR or EPDR1 siRNA. (**D**) Densitometric quantification normalized to β-actin from six independent experiments.

Silencing of EPDR1 in IPF lung fibroblasts using siRNA resulted in a marked decrease in SA-β-gal staining, a well-established marker of cellular senescence (Figure 6A). Western blot analysis demonstrated that knockdown of EPDR1 led to a significant reduction in the expression levels of LC3B [1.25 (1.19–2.00) vs. 0.74 (0.61–0.97) AU], p62 [0.84 (0.0.48–1.20) vs. 0.37 (0.14–0.48) AU], p21 [1.11 (0.89–1.76) vs. 0.64 (0.36–0.99) AU], and p16 [1.31 (0.98–1.43) vs. 0.62 (0.43–0.66) AU] compared to cells treated with SCR. In contrast, LAMP1 expression remained largely unchanged [1.17 (0.92–1.71) vs. 1.30 (0.94–1.79) AU], indicating that EPDR1 suppression did not affect overall lysosomal content (Figure 6B,C).

## 4. Discussion

In this study, we identified EPDR1 as a novel regulator enriched in IPF fibroblasts and fibrotic lung tissues. EPDR1 levels in BALF and serum were associated with disease severity and poor prognosis, highlighting its potential as a biomarker. Mechanistically, EPDR1 localized to lysosomes, where it impaired autophagic flux and lysosomal acidification. Suppression of EPDR1 alleviated fibroblast senescence and restored proteostasis, supporting its role as a driver of fibrotic remodeling through disruption of lysosomal homeostasis.

Consistent with this concept, EPDR1 was markedly upregulated in IPF fibroblasts and within fibrotic lesions, where it co-localized with myofibroblast markers (α-SMA, COL1A1). Its parallel elevation in BALF and serum suggests that EPDR1 dysregulation extends beyond fibrotic foci and reflects a systemic signature of fibroblast dysfunction. This dual significance underscores EPDR1 as both a mechanistic contributor to fibrosis and a clinically measurable biomarker. The cellular origin of extracellular EPDR1 remains to be clarified. While not directly assessed in our study, the strong expression of EPDR1 in fibroblasts within fibrotic lesions suggests a mesenchymal contribution to EPDR1 accumulation in local (lung) and systemic compartments. Supporting this interpretation, multiple single-cell transcriptomic datasets consistently demonstrate increased EPDR1 expression in fibroblasts and myofibroblasts from IPF lungs [24,25,26,27]. Furthermore, elevated EPDR1 expression in alveolar type I epithelial cells [24,26] indicates that both mesenchymal and epithelial compartments may contribute to EPDR1 upregulation in fibrotic lungs. From a clinical perspective, soluble biomarkers that reflect fibrotic activity and predict outcomes are increasingly important in IPF management [28,29]. Here, we demonstrate that BALF EPDR1 not only discriminates IPF diagnosis but also predicts survival. The higher sensitivity of BALF compared with serum likely reflects its direct representation of the local fibrotic microenvironment in the lung, whereas systemic dilution and clearance may attenuate the sensitivity of serum measurements despite comparable specificity. Serum EPDR1 also carried prognostic significance, though to a lesser extent. These findings suggest that EPDR1 levels in accessible fluids mirror fibrotic burden and may provide a noninvasive tool for disease monitoring.

The pathological relevance of EPDR1 is not confined to pulmonary fibrosis. Elevated EPDR1 has been implicated in multiple cancers, where it correlates with advanced stage, metastasis, and poor survival [30,31,32]. Mechanistically, cancer studies suggest that EPDR1 promotes adhesion, migration, and epithelial–mesenchymal transition in malignant epithelial cells, while stromal and immune cells also contribute to its expression [31,33]. Taken together, these observations suggest that EPDR1 functions as a conserved marker of aggressive pathology across both fibrotic and malignant contexts.

**Figure 7:**
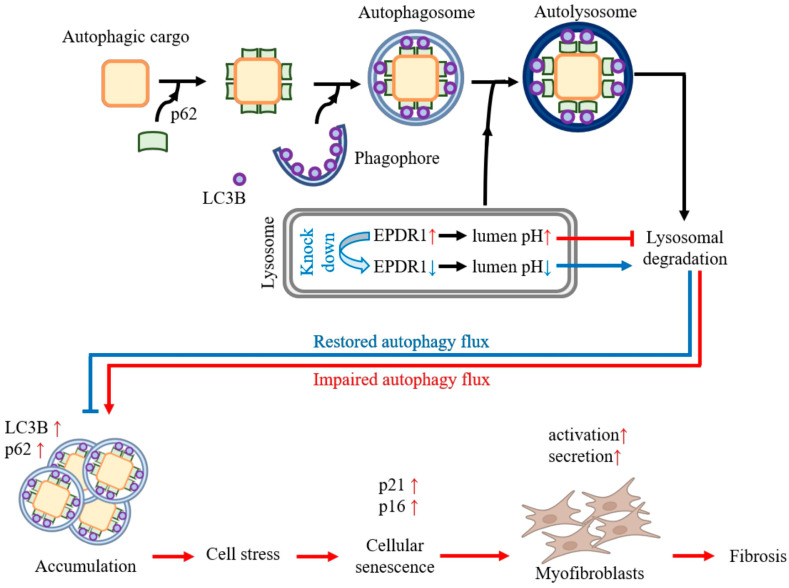
Schematic model of EPDR1-mediated regulation of autophagy in IPF lung fibroblasts. EPDR1 functions as a negative regulator of lysosomal acidification, leading to impaired autophagic flux and enhanced fibroblast senescence in IPF. Silencing of EPDR1 restores lysosomal activity and autophagy, thereby reducing cellular senescence and proteostasis stress. This EPDR1-driven lysosomal–autophagic dysfunction provides a mechanistic link between aberrant fibroblast activation and fibrotic progression, positioning EPDR1 as a critical mediator of maladaptive mesenchymal reprogramming in IPF.

Autophagy and senescence are central to fibroblast dysfunction in IPF [34,35,36]. Impaired autophagy limits clearance of damaged organelles, while senescent fibroblasts drive persistent ECM deposition and profibrotic signaling [34,36,37]. EPDR1, a lysosome-associated type II transmembrane glycoprotein structurally related to protocadherins and ependymins [38], appears to converge on these maladaptive processes. We observed reduced LysoTracker intensity in IPF fibroblasts, consistent with defective lysosomal acidification. EPDR1 expression correlated with accumulation of autophagy- and senescence-associated markers (p62, p21, p16), while EPDR1 knockdown restored lysosomal acidification, enhanced autophagic flux, and reduced senescence. The concomitant increase in LC3B and p62 is consistent with impaired autophagy flux rather than enhanced autophagy activity, as both proteins are normally degraded via lysosomal processing under intact flux [39]. This interpretation aligns with studies in IPF lung fibroblasts and tissues [34,40], senescence models showing flux blockade with LC3/p62 accumulation [41], and pathological analyses supporting marker-based flux interpretation [42], and is further supported by a recent review in IPF [43]. These findings suggest that EPDR1 may act as a negative regulator of lysosomal acidification, thereby contributing to impaired autophagy and fibroblast senescence in IPF (Figure 7). However, the precise molecular mechanism remains to be fully elucidated, and additional studies, such as co-immunoprecipitation or lipid-binding assays, will be required to define how EPDR1 regulates lysosomal activity. Collectively, these data position EPDR1 as a nodal effector within maladaptive mesenchymal reprogramming, linking lysosomal dysfunction, cellular senescence, and progressive fibrosis. Our results align with accumulating evidence that lysosomal impairment constitutes a central driver of fibrosis, providing a broader biological framework for EPDR1-mediated pathogenicity.

This study has several limitations. First, the effects of EPDR1 on non-fibroblast populations, including epithelial and immune cells, remain to be investigated. Second, although we identified significant associations between EPDR1 levels and clinical outcomes, validation in independent cohorts is needed. Third, mechanistic experiments were restricted to patient-derived primary fibroblasts, and in vivo studies employing genetic or pharmacologic EPDR1 modulation in animal models will be necessary to confirm its pathogenic role. Fourth, expression analyses of autophagy- and senescence-related markers (LAMP1, LC3B, p62, p21, and p16) were limited to Western blotting of primary fibroblasts due to the limited availability of lung tissue samples. In situ validation using tissue specimens will be necessary in future studies. Finally, although EPDR1 knockdown was shown to improve lysosomal function and reduce senescence markers, the direct mechanism underlying these effects remains unclear. Additional mechanistic experiments, such as co-immunoprecipitation or lipid-binding assays, will be required to delineate how EPDR1 regulates lysosomal activity.

## 5. Conclusions

Our findings identify EPDR1 as a novel regulator of fibroblast dysfunction in IPF, acting through lysosomal impairment and promotion of cellular senescence. Elevated EPDR1 expression in fibrotic lungs and biological fluids, together with its association with disease severity and prognosis, underscores its translational relevance as both a mechanistic driver and a potential biomarker. Targeting EPDR1 may offer a new therapeutic avenue to restore cellular homeostasis and mitigate fibrotic remodeling in progressive interstitial lung diseases.

## Figures and Tables

**Table 1:** Clinical characteristics of the study subjects who underwent bronchoalveolar lavage.

Clinical Parameters	Control	IPF
No.	52	131
Age (year)	52 (44–62)	65 (59–72)
Sex (male/female)	34/18	86/45
Smoke (CS/ES/NS)	28/11/13	58/45/28
Survival/death	ND	79/52
Follow up duration (years)	ND	4.77 (1.64–7.46)
FVC (% pred.)	87.26 ± 10.26	73.6 ± 16.77 *#
DL_CO_ (% pred.)	88.4 ± 19.75	70.11 ± 20.98 *#
BAL total cell count (×10^5^)	0.81 ± 0.8	5.63 ± 5.92 *
Macrophages (%)	94.49 ± 3.53	63.77 ± 25.17 *
Neutrophils (%)	2.55 ± 2.58	28.05 ± 25.19 *
Eosinophils (%)	0.42 ± 0.82	3.2 ± 6.94 *
Lymphocytes (%)	2.54 ± 2	4.98 ± 5.18 *

Serum EPDR1 levels were measured in 45 of 52 controls and 114 of 131 IPF patients. IPF: idiopathic pulmonary fibrosis, CS/ES/NS: current-smokers/ex-smokers/never-smokers, ND: not determined, FVC: forced vital capacity, DL_CO_: diffusing capacity of the lung for carbon monoxide. Values are presented as median (interquartile range) or mean ± standard error of the mean. Statistical significance: * *p* < 0.05 vs. control in BALF; # *p* < 0.05 vs. control in serum.

**Table 2:** Cox proportional hazards model of risk factors for mortality in IPF.

Parameter	HR	95% CI	*p*-Value
Univariate analysis			
Age (year)	1.01	0.97–1.04	0.605
Sex (male vs. female)	0.71	0.35–1.43	0.343
Smoke (yes vs. none)	1.69	0.83–3.41	0.143
BMI, kg/m^2^	1.01	0.89–1.15	0.849
FVC (% pred)	0.96	0.94–0.98	0.001
DL_CO_ (% pred)	0.97	0.95–0.99	0.006
BALF EPDR1 ≥ 1190.2 ng/mL	2.19	1.08–4.43	0.029
Serum EPDR1 ≥ 240.5 ng/mL	2.65	1.29–5.45	0.008
Multivariate analysis			
BALF EPDR1 ≥ 1190.2 ng/mL	2.21	1.04–4.67	0.038
Serum EPDR1 ≥ 240.5 ng/mL	2.61	1.13–5.95	0.034

HR, hazard ratio; 95% CI, 95% confidence interval; BMI, body mass index; FVC, forced vital capacity; DL_CO_, diffusing capacity for carbon monoxide.

## Data Availability

The data presented in this study are available in the article.

## Supplementary Materials

The following supporting information can be downloaded at: https://www.mdpi.com/article/10.3390/cells14191515/s1, Figure S1: Western blot validation of cell-type-specific markers in cultured primary lung fibroblasts. Cell lysates were analyzed by western blotting for fibroblast purity using the following cell-type-specific markers: E-cadherin (epithelial), CD31 (endothelial), CD45 (hematopoietic), and vimentin (fibroblast). β-actin was used as a loading control. The primary antibodies used were: anti-E-cadherin (Invitrogen, 33-4000, 1:1000), anti-CD31 (Santa Cruz, sc-376764, 1:1000), anti-CD45 (Santa Cruz, sc-1178, 1:1000), anti-vimentin (Santa Cruz, sc-6260, 1:2000), and anti-β-actin (Sigma-Aldrich, A1978, 1:50,000); Figure S2: mRNA expression of lyso-some-related genes in primary lung fibroblasts from controls and IPF patients. Quantitative PCR was performed in fibroblasts from controls (*n* = 10) and IPF patients (*n* = 10). The analyzed gene panel included markers of lysosomal acidification machinery (ATP6V1A, ATP6V1B2, ATP6V0C, ATP6V0D1) and lysosomal proteases (CTSB, CTSD). Data from six independent experiments are presented as box-and-whisker plots. Statistical significance was assessed using the Mann–Whitney U test. Table S1: Clinical characteristics of the study subjects who underwent fibroblasts culture; Table S2: List of primer sequences.

## References

[1] Moss B.J., Ryter S.W., Rosas I.O. (2022). Pathogenic Mechanisms Underlying Idiopathic Pulmonary Fibrosis. Annu. Rev. Pathol..

[2] Herrera J., Henke C.A., Bitterman P.B. (2018). Extracellular matrix as a driver of progressive fibrosis. J. Clin. Investig..

[3] Liu T., Gonzalez De Los Santos F., Hirsch M., Wu Z., Phan S.H. (2021). Noncanonical Wnt Signaling Promotes Myofibroblast Differentiation in Pulmonary Fibrosis. Am. J. Respir. Cell Mol. Biol..

[4] Jin C., Chen Y., Wang Y., Li J., Liang J., Zheng S., Zhang L., Li Q., Wang Y., Ling F. (2024). Single-cell RNA sequencing reveals special basal cells and fibroblasts in idiopathic pulmonary fibrosis. Sci. Rep..

[5] Hanmandlu A., Zhu L., Mertens T.C.J., Collum S., Bi W., Xiong F., Wang R., Amirthalingam R.T., Ren D., Han L. (2022). Transcriptomic and Epigenetic Profiling of Fibroblasts in Idiopathic Pulmonary Fibrosis. Am. J. Respir. Cell Mol. Biol..

[6] Wu S., Liu M., Zhang M., Ye X., Gu H., Jiang C., Zhu H., Ye X., Li Q., Huang X. (2024). The gene expression of CALD1, CDH2, and POSTN in fibroblast are related to idiopathic pulmonary fibrosis. Front. Immunol..

[7] Lee J.U., Cheong H.S., Shim E.Y., Bae D.J., Chang H.S., Uh S.T., Kim Y.H., Park J.S., Lee B., Shin H.D. (2017). Gene profile of fibroblasts identify relation of CCL8 with idiopathic pulmonary fibrosis. Respir. Res..

[8] Tsukui T., Sun K.H., Wetter J.B., Wilson-Kanamori J.R., Hazelwood L.A., Henderson N.C., Adams T.S., Schupp J.C., Poli S.D., Rosas I.O. (2020). Collagen-producing lung cell atlas identifies multiple subsets with distinct localization and relevance to fibrosis. Nat. Commun..

[9] Staats K.A., Wu T., Gan B.S., O’Gorman D.B., Ophoff R.A. (2016). Dupuytren’s disease susceptibility gene, EPDR1, is involved in myofibroblast contractility. J. Dermatol. Sci..

[10] Della Valle M.C., Sleat D.E., Sohar I., Wen T., Pintar J.E., Jadot M., Lobel P. (2006). Demonstration of lysosomal localization for the mammalian ependymin-related protein using classical approaches combined with a novel density shift method. J. Biol. Chem..

[11] Wei Y., Xiong Z.J., Li J., Zou C., Cairo C.W., Klassen J.S., Privé G.G. (2019). Crystal structures of human lysosomal EPDR1 reveal homology with the superfamily of bacterial lipoprotein transporters. Commun. Biol..

[12] Takaya K., Asou T., Kishi K. (2022). Aging Fibroblasts Adversely Affect Extracellular Matrix Formation via the Senescent Humoral Factor Ependymin-Related Protein 1. Cells.

[13] Kolter T., Sandhoff K. (2010). Lysosomal degradation of membrane lipids. FEBS Lett..

[14] Schulze H., Sandhoff K. (2014). Sphingolipids and lysosomal pathologies. Biochim. Biophys. Acta.

[15] Raghu G., Collard H.R., Egan J.J., Martinez F.J., Behr J., Brown K.K., Colby T.V., Cordier J.F., Flaherty K.R., Lasky J.A. (2011). An official ATS/ERS/JRS/ALAT statement: Idiopathic pulmonary fibrosis: Evidence-based guidelines for diagnosis and management. Am. J. Respir. Crit. Care Med..

[16] Raghu G., Remy-Jardin M., Myers J.L., Richeldi L., Ryerson C.J., Lederer D.J., Behr J., Cottin V., Danoff S.K., Morell F. (2018). Diagnosis of Idiopathic Pulmonary Fibrosis. An Official ATS/ERS/JRS/ALAT Clinical Practice Guideline. Am. J. Respir. Crit. Care Med..

[17] Livak K.J., Schmittgen T.D. (2001). Analysis of relative gene expression data using real-time quantitative PCR and the 2(-Delta Delta C(T)) Method. Methods.

[18] Park C.S., Chung S.W., Ki S.Y., Lim G.I., Uh S.T., Kim Y.H., Choi D.I., Park J.S., Lee D.W., Kitaichi M. (2000). Increased levels of interleukin-6 are associated with lymphocytosis in bronchoalveolar lavage fluids of idiopathic nonspecific interstitial pneumonia. Am. J. Respir. Crit. Care Med..

[19] Adar Y., Stark M., Bram E.E., Nowak-Sliwinska P., van den Bergh H., Szewczyk G., Sarna T., Skladanowski A., Griffioen A.W., Assaraf Y.G. (2012). Imidazoacridinone-dependent lysosomal photodestruction: A pharmacological Trojan horse approach to eradicate multidrug-resistant cancers. Cell Death Dis..

[20] Fang C., Weng T., Hu S., Yuan Z., Xiong H., Huang B., Cai Y., Li L., Fu X. (2021). IFN-γ-induced ER stress impairs autophagy and triggers apoptosis in lung cancer cells. Oncoimmunology.

[21] Shakhov A.S., Kovaleva P.A., Churkina A.S., Kireev I.I., Alieva I.B. (2022). Colocalization Analysis of Cytoplasmic Actin Isoforms Distribution in Endothelial Cells. Biomedicines.

[22] Álvarez D., Cárdenes N., Sellarés J., Bueno M., Corey C., Hanumanthu V.S., Peng Y., D’Cunha H., Sembrat J., Nouraie M. (2017). IPF lung fibroblasts have a senescent phenotype. Am. J. Physiol. Lung Cell Mol. Physiol..

[23] DeLong E.R., DeLong D.M., Clarke-Pearson D.L. (1988). Comparing the areas under two or more correlated receiver operating characteristic curves: A nonparametric approach. Biometrics.

[24] Adams T.S., Schupp J.C., Poli S., Ayaub E.A., Neumark N., Ahangari F., Chu S.G., Raby B.A., DeIuliis G., Januszyk M. (2020). Single-cell RNA-seq reveals ectopic and aberrant lung-resident cell populations in idiopathic pulmonary fibrosis. Sci. Adv..

[25] Habermann A.C., Gutierrez A.J., Bui L.T., Yahn S.L., Winters N.I., Calvi C.L., Peter L., Chung M.I., Taylor C.J., Jetter C. (2020). Single-cell RNA sequencing reveals profibrotic roles of distinct epithelial and mesenchymal lineages in pulmonary fibrosis. Sci. Adv..

[26] Reyfman P.A., Walter J.M., Joshi N., Anekalla K.R., McQuattie-Pimentel A.C., Chiu S., Fernandez R., Akbarpour M., Chen C.I., Ren Z. (2019). Single-Cell Transcriptomic Analysis of Human Lung Provides Insights into the Pathobiology of Pulmonary Fibrosis. Am. J. Respir. Crit. Care Med..

[27] Morse C., Tabib T., Sembrat J., Buschur K.L., Bittar H.T., Valenzi E., Jiang Y., Kass D.J., Gibson K., Chen W. (2019). Proliferating SPP1/MERTK-expressing macrophages in idiopathic pulmonary fibrosis. Eur. Respir. J..

[28] Maher T.M., Strek M.E. (2019). Antifibrotic therapy for idiopathic pulmonary fibrosis: Time to treat. Respir. Res..

[29] D’Agnano V., Mariniello D.F., Ruotolo M., Quarcio G., Moriello A., Conte S., Sorrentino A., Sanduzzi Zamparelli S., Bianco A., Perrotta F. (2024). Targeting Progression in Pulmonary Fibrosis: An Overview of Underlying Mechanisms, Molecular Biomarkers, and Therapeutic Intervention. Life.

[30] Yang Y., Xu H., Zhu H., Yuan D., Zhang H., Liu Z., Zhao F., Liang G. (2022). EPDR1 levels and tumor budding predict and affect the prognosis of bladder carcinoma. Front. Oncol..

[31] Gimeno-Valiente F., Riffo-Campos Á.L., Ayala G., Tarazona N., Gambardella V., Rodríguez F.M., Huerta M., Martínez-Ciarpaglini C., Montón-Bueno J., Roselló S. (2020). EPDR1 up-regulation in human colorectal cancer is related to staging and favours cell proliferation and invasiveness. Sci. Rep..

[32] Chen R., Zhang Y. (2020). EPDR1 correlates with immune cell infiltration in hepatocellular carcinoma and can be used as a prognostic biomarker. J. Cell Mol. Med..

[33] Min H.Y., Sim J.Y., Ahn J.H., Kang N.W., Boo H.J., Kim J., Yu N.Y., Bottacin M., Huh J., Park C.S. (2025). Gaylussacin, a stilbene glycoside, inhibits chronic obstructive pulmonary disease in mice. Redox Biol..

[34] Patel A.S., Lin L., Geyer A., Haspel J.A., An C.H., Cao J., Rosas I.O., Morse D. (2012). Autophagy in idiopathic pulmonary fibrosis. PLoS ONE.

[35] Lehmann M., Korfei M., Mutze K., Klee S., Skronska-Wasek W., Alsafadi H.N., Ota C., Costa R., Schiller H.B., Lindner M. (2017). Senolytic drugs target alveolar epithelial cell function and attenuate experimental lung fibrosis ex vivo. Eur. Respir. J..

[36] Schafer M.J., White T.A., Iijima K., Haak A.J., Ligresti G., Atkinson E.J., Oberg A.L., Birch J., Salmonowicz H., Zhu Y. (2017). Cellular senescence mediates fibrotic pulmonary disease. Nat. Commun..

[37] Lin Y., Xu Z. (2020). Fibroblast Senescence in Idiopathic Pulmonary Fibrosis. Front. Cell Dev. Biol..

[38] Ahmed T., Flores P.C., Pan C.C., Ortiz H.R., Lee Y.S., Langlais P.R., Mythreye K., Lee N.Y. (2022). EPDR1 is a noncanonical effector of insulin-mediated angiogenesis regulated by an endothelial-specific TGF-β receptor complex. J. Biol. Chem..

[39] Kimura S., Noda T., Yoshimori T. (2007). Dissection of the autophagosome maturation process by a novel reporter protein, tandem fluorescent-tagged LC3. Autophagy.

[40] Araya J., Kojima J., Takasaka N., Ito S., Fujii S., Hara H., Yanagisawa H., Kobayashi K., Tsurushige C., Kawaishi M. (2013). Insufficient autophagy in idiopathic pulmonary fibrosis. Am. J. Physiol. Lung Cell Mol. Physiol..

[41] Tai H., Wang Z., Gong H., Han X., Zhou J., Wang X., Wei X., Ding Y., Huang N., Qin J. (2017). Autophagy impairment with lysosomal and mitochondrial dysfunction is an important characteristic of oxidative stress-induced senescence. Autophagy.

[42] Langer R., Neppl C., Keller M.D., Schmid R.A., Tschan M.P., Berezowska S. (2018). Expression Analysis of Autophagy Related Markers LC3B, p62 and HMGB1 Indicate an Autophagy-Independent Negative Prognostic Impact of High p62 Expression in Pulmonary Squamous Cell Carcinomas. Cancers.

[43] Yue Y.L., Zhang M.Y., Liu J.Y., Fang L.J., Qu Y.Q. (2022). The role of autophagy in idiopathic pulmonary fibrosis: From mechanisms to therapies. Ther. Adv. Respir. Dis..

