# Supplementary material for: EPDR1 Links Fibroblast Dysfunction to Disease Severity in Idiopathic Pulmonary Fibrosis
Source: Cells. 2025 Sep 28;14(19):1515. doi: 10.3390/cells14191515 (PMC12523719; doi:10.3390/cells14191515)
Supplement: Supplementary file 1 [file cells-14-01515-s001.zip › cells-3873122-supplementary.pdf]

Table S1. Clinical characteristics of the study subjects who underwent fibroblasts culture

Variables	Control	IPF
No.	10	10
Age (year)	54(46-74)	61(50-72)
Sex (male/female)	3/7	4/6
Smoke (CS/ES/NS)	2/1/7	3/3/4
Survival/Death	ND	6/4
Follow up duration (years)	ND	4.2(2.9-7.0)
FVC (% pred.)	98(77-106)	84(47-104) *
FEV1 (% pred.)	103.3(98-118)	94.9(81.8-108)
DL_{CO} (% pred.)	88(71-120)	68(39-90) *

Fibroblasts culture: Lung fibroblasts were cultured from normal lungs of 10 subjects (Control) who underwent surgery to remove localized lung cancer and those from surgical biopsy specimens of 10 patients with IPF (IPF). CS /ES/NS: current-smokers/ex-smokers/never-smokers, ND: not determined. Data are shown as median (interquartile range: IQR). Differences between the controls and the subjects with IPF were compared using Mann-Whitney U-test. * Compared with controls, $P < 0.05$

Table S2. **List of primer sequences**

Genes	Forward (5' to 3')	Reverse (5' to 3')
EPDR1	GAGAGGAAGGCGCTGATCC	TGGCTTGGTCAATCTGAAACA
LAMP1	AGACACACACCTTTTCCCCA	GAAAGGTACGCCTGGATGGT
ATP6V1A	CCCCAGAGGAGTAAACGTGTC	TTCCTCTGTTTCGTGGGGGT
ATP6V1B2	AGCTGAATTTCTGGCGTACCA	AATCGAGCCGTTTCTCCCTT
ATP6V1E1	CAACATAGAGAAAGGTCGGCT	TCTTGCTCTGAGGACTTTGAGT
ATP6V1F	AGTGATCGGAGACGAGGACA	GGATGGACTGCTGGTGGG
ATP6V0A1	CAAAGTGCGGAAGATGAAGGC	GTTGGGGGAGTCTGGTTTGTC
CTSB	AGTGTACCAACACGTCACCG	TCCAGCCACCACTTCTGATTC
CTSD	ATCTCCGTCAACAACGTGCT	TGCGGGTGACATTCAGGTAG
CTSL	TCAGGCAGGTGATGAATGGC	GCCCAACAAGAACCACACTG
CTSS	GCACTCATACGATCTGGGCA	TGGGAACTCTCAGGGAACTCA
CTSZ	ACCAGCACATCCCCCAATAC	TTGTTGCAGGTCTCGTCAGG
TFEB	AATACCCCCGTCCACTTCCA	ACCTTCTGATGCTGCGACTG
α-SMA	ACTGGGACGACATGGAAAAG	TACATGGCTGGGACATTGAA
COL1A1	ACGAAGACATCCCACCAATCACCT	AGATCACGTCATCGCACAACACCT
Fibronectin	ACAACACCGAGGTGACTGAGAC	GGACACAACGATGCTTCCTGAG
β-actin	GGACTTCGAGCAAGAGATGG	AGCACTGTGTTGGCGTACAG

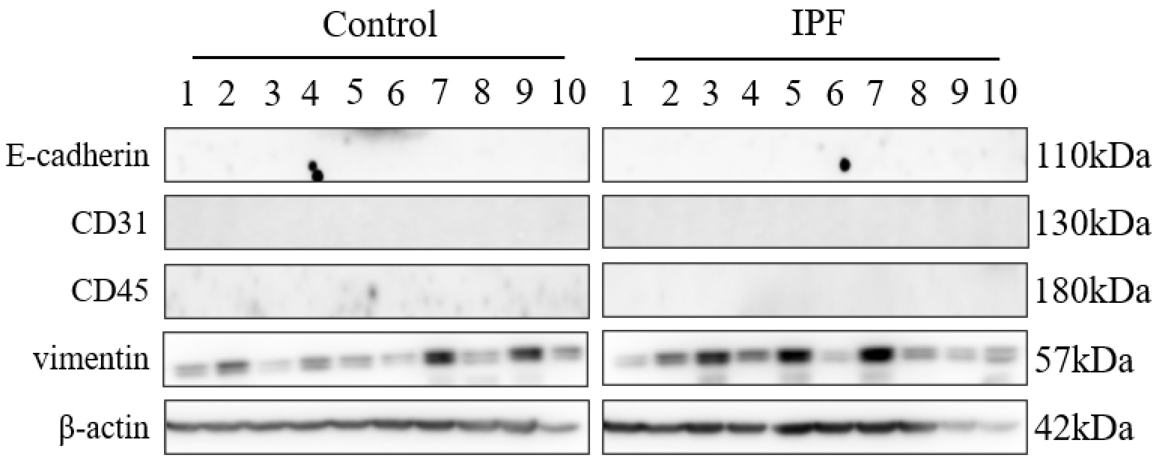

Figure S1. Western blot validation of cell-type-specific markers in cultured primary lung fibroblasts. Cell lysates were analyzed by western blotting for fibroblast purity using the following cell-type-specific markers: E-cadherin (epithelial), CD31 (endothelial), CD45 (hematopoietic), and vimentin (fibroblast). β-actin was used as a loading control. The primary antibodies used were: anti-E-cadherin (Invitrogen, 33-4000, 1:1000), anti-CD31 (Santa Cruz, sc-376764, 1:1000), anti-CD45 (Santa Cruz, sc-1178, 1:1000), anti-vimentin (Santa Cruz, sc-6260, 1:2000), and anti-β-actin (Sigma-Aldrich, A1978, 1:50000).

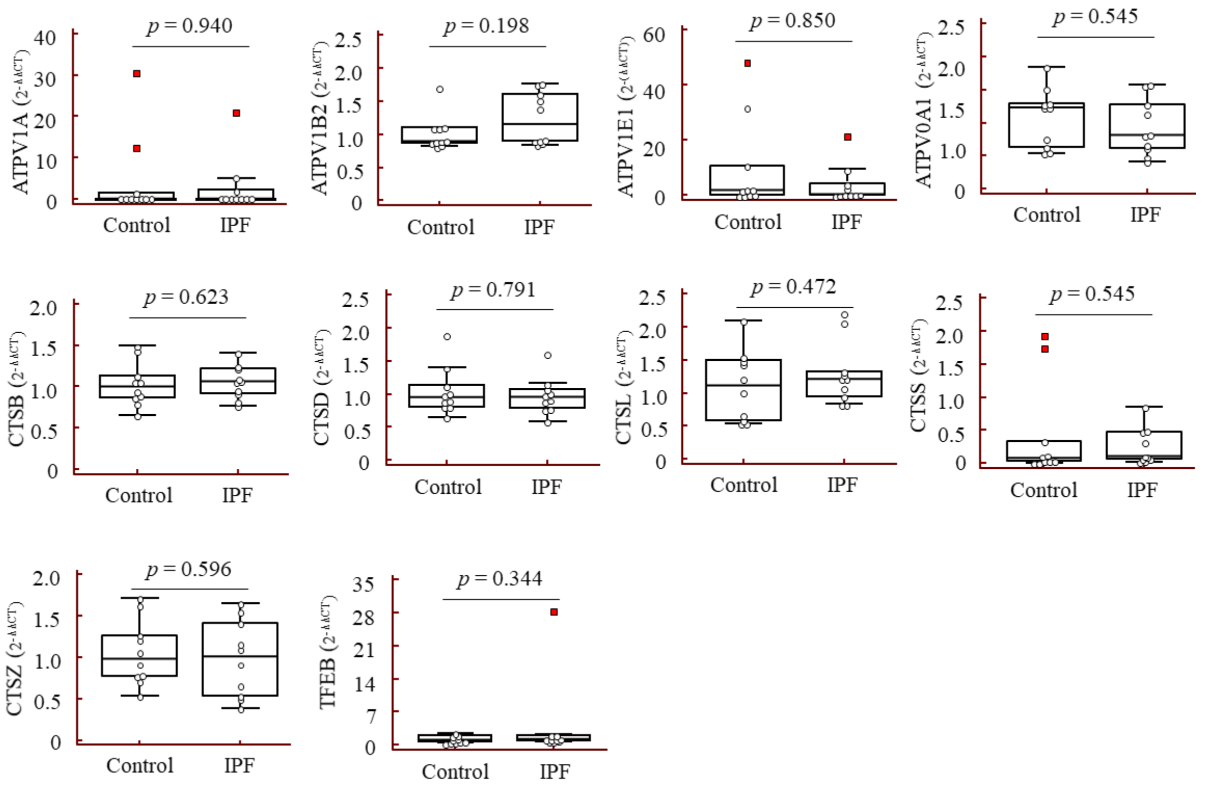

Figure S2. mRNA expression of lysosome-related genes in primary lung fibroblasts from controls and IPF patients. Quantitative PCR was performed in fibroblasts from controls (n = 10) and IPF patients (n = 10). The analyzed gene panel included markers of lysosomal acidification machinery (ATP6V1A, ATP6V1B2, ATP6V0C, ATP6V0D1) and lysosomal proteases (CTSB, CTSD). Data from six independent experiments are presented as box-and-whisker plots. Statistical significance was assessed using the Mann–Whitney U test.
